# Orientation of the Mitotic Spindle in Blood Vessel Development

**DOI:** 10.3389/fcell.2020.583325

**Published:** 2020-09-17

**Authors:** Xuemei Wu, Jun Zhou, Dengwen Li

**Affiliations:** ^1^State Key Laboratory of Medicinal Chemical Biology, College of Life Sciences, Nankai University, Tianjin, China; ^2^Shandong Provincial Key Laboratory of Animal Resistance Biology, Collaborative Innovation Center of Cell Biology in Universities of Shandong, College of Life Sciences, Institute of Biomedical Sciences, Shandong Normal University, Jinan, China

**Keywords:** spindle orientation, angiogenesis, mitotic spindle, cell proliferation, blood vessel development

## Abstract

Angiogenesis requires coordinated endothelial cell specification, proliferation, and collective migration. The orientation of endothelial cell division is tightly regulated during the earliest stages of blood vessel formation in response to morphogenetic cues and the controlled orientation of the mitotic spindle. Consequently, oriented cell division is a vital mechanism in vessel morphogenesis, and defective spindle orientation can perturb the spatial arrangement of daughter cells and consequently contribute to several diseases related to vascular development. Many factors affect endothelial cell proliferation and orientation and therefore blood vessel formation, with the relationship between improper spindle orientation in endothelial cells and various diseases extensively studied. Here we review the molecular mechanisms driving the orientation of endothelial cell division, particularly with respect to the mitotic spindle, and how these processes affect vascular development, disease pathogenesis, and their potential as novel targets.

## Introduction

Blood vessel development, which includes vasculogenesis and angiogenesis, is crucial for the formation of the cardiovascular system and blood vessel regeneration after injury. The vasculature is one of the first organ systems to develop during vertebrate embryogenesis (Wilkinson and van Eeden, 2014). Angiogenesis is important in a number of pathophysiological processes (Sajib et al., 2018), not only supporting the developing embryo and in wound healing but also in many diseases including cancer, infectious arthritis, and psoriasis (Carmeliet, 2000; Sajib et al., 2018). The formation of the vascular plexus requires exquisite regulation and integration of several cellular processes: endothelial cells sprout in response to morphogenetic cues and must actively divide to expand the endothelial cell pool. As a consequence, abnormal blood vessel development contributes to numerous diseases such as cancer and intraocular vascular disorders (Apte et al., 2019), with aberrant endothelial cell proliferation, migration, polarity, and the maintenance of intercellular junctions central processes (Hogan and Schulte-Merker, 2017).

**FIGURE 1:**
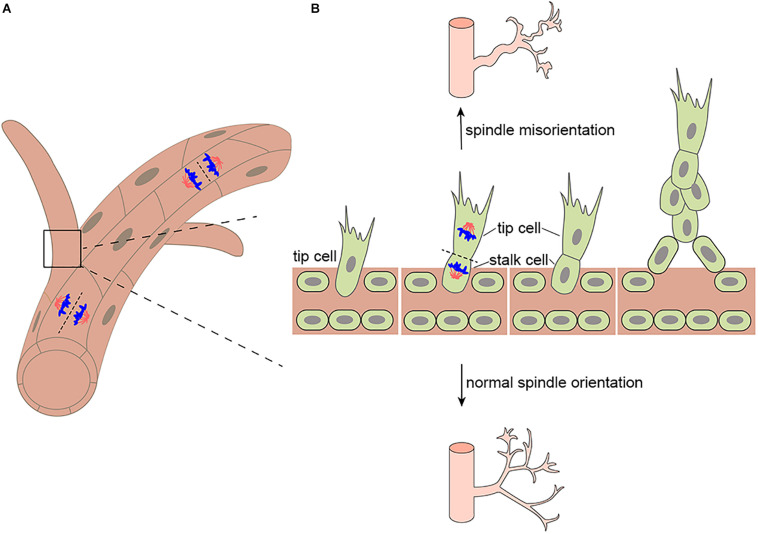
Mitotic spindle orientation in endothelial cell affects vessel morphogenesis. **(A)** Endothelial divisions oriented perpendicular to the vessel long axis (on top) would effectively lengthen the vessel, whereas divisions oriented parallel to the vessel long axis (on bottom) would effectively increase the vessel diameter. **(B)** During branching morphogenesis, highly motile endothelial tip cells sprout from parental vessels and lead stalk cells. Upon tip cell division, the mitotic spindle is displaced to the proximal pole of the cell before anaphase. This introduces cell size asymmetry and generates daughter cells with distinct VEGF signaling levels. In addition, aberrant mitotic spindle orientation changes endothelial cell cleavage plane and further increasing vessel tortuosity and dilation.

Spindle orientation determines the fate and position of daughter cells during mitosis (Li J. et al., 2019) and plays an important role in development, including epithelium and vascular endothelium development. Epithelium development plays a critical role in organ development and tissue repair (Ting Song, 2020), which requires proper orientation of the mitotic spindle (Xie and Zhou, 2017; Xie et al., 2017). In proliferating epithelium, planar cell division occurs by orienting mitotic spindles into the epithelial plane to ensure organized tissue formation (Luo et al., 2016; Nakajima, 2018). Asymmetric positioning of the mitotic spindle during endothelial tip cell division generates multicellular polarity, which drives coordinated and collective cell migration in angiogenesis (Costa et al., 2016). During asymmetric division, mitotic spindles must be placed on the polarization axis to ensure the correct orientation of daughter cells (Liro and Rose, 2016). In view of the direct and intimate connection between spindle orientation and endothelial cell division and polarization, spindle orientation during mitosis is essential to blood vessel development, so, when abnormal, the proteins and processes related to abnormal spindle orientation might be expected to participate in vascular development diseases (Zhong and Zhou, 2017; Figure 1). In this review, we summarize how spindle orientation regulates endothelial cell division to affect vascular development and discuss the relationship between misorientation and pathological state.

## Blood Vessel Development

Oxygen and nutrient transport in developing embryos depends on the formation of vascular networks (Ma and Zhou, 2020), and many pathologies involve blood vessel development and remodeling (Potente et al., 2011). Each organ in the human body has its own capillary bed with both general and specific functions to respond to dynamic systemic and local changes (Augustin and Koh, 2017). Blood vessel development includes vasculogenesis and angiogenesis. Vasculogenesis, the formation of embryonic blood vessels, involves the differentiation, migration, and coalescence of angioblasts and the polarization of endothelial cells to form a vascular lumen and create a primordial vascular network. During angiogenesis, new blood vessels are formed from existing capillaries or venules by endothelial cell proliferation, differentiation, and migration (Carmeliet and Jain, 2011). When endothelial cells form sprouts, two distinct phenotypes are undertaken by the endothelial cells asymmetric division in the nascent blood vessel sprout, namely the tip cell phenotype and the stalk cell phenotype (Koon et al., 2018). Tip cells bring about motile behavior which migrate toward the angiogenic source upon stimulation by chemotactic factors. Stalk cells trail behind the tip cells to support the growth of the vessel by their proliferative capacity (Gerhardt et al., 2003; Figure 1B). In addition, stalk cells ensure stability and integrity of the young sprout by forming adherent and tight junctions (Blanco and Gerhardt, 2013). Therefore, its dysfunction can cause inflammatory, infectious and immune disorders (Carmeliet, 2003). Furthermore, polarized positioning of the mitotic spindle functions to generate intrinsically asymmetric daughters of tip cell division, which is essential for vessel sprout formation. Many aspects in tissue morphogenesis are attributed to a collective behavior of the participating cells (Lv et al., 2020). Daughters of tip cell rapidly self-organize into leading and trailing cells following division, which maintains uninterrupted collective migration during vessel proliferation (Costa et al., 2016). During vascular development, endothelial cell migration, proliferation, polarity, differentiation, and intercellular communication must be tightly coordinated for functional vascular morphogenesis (Herbert and Stainier, 2011). Blood vessel development plays an important role in vascular barrier formation (Tam and Watts, 2010), tumorigenesis (Yadav et al., 2015), and ischemic, inflammatory, infectious, and immune disorders (Carmeliet, 2003).

## The Role of Spindle Orientation in Blood Vessel Development

The correct separation of chromosomes into daughter cells of different sizes or cell fates requires precise spindle orientation to control cell fate choices, tissue architecture, and tissue morphogenesis (Morin and Bellaïche, 2011; di Pietro et al., 2016; Chen et al., 2020). Blood vessel development depends on vascular lumen formation, which requires the precise mitotic spindle orientation of endothelial cells and the establishment of polarity to form opposed apical cell surfaces (Neufeld et al., 2014). Endothelial cleavage plane oriented perpendicular to the blood vessel long axis would effectively lengthen the blood vessel, whereas divisions oriented parallel to the blood vessel long axis would effectively increase the blood vessel diameter (Figure 1A). In particular, during angiogenesis, the asymmetric division of endothelial tip cells generates heterogeneous daughter cells that maintain hierarchical tip-stalk organization and synchronize collective movements (Costa et al., 2016). These processes are closely related to mitotic spindle orientation, which is precisely controlled by many cues, either intrinsic or extrinsic, such as natural direct current electric fields (DC-EFs) and many signaling pathways and proteins that play an important role in spindle orientation including VEGF signaling, the Rho family of GTPases Cdc42 are also indispensable to vascular development and regeneration. Highlighting its importance in blood vessel development, the aberrant regulation of spindle orientation has been linked to a variety of human diseases.

### VEGF Signaling

Among the already identified pro-angiogenic molecules, vascular endothelial growth factor (VEGF) is established as the key angiogenic growth factor (Melincovici et al., 2018; Li S. et al., 2019), by regulating blood vessel growth and maintenance. Pioneering studies showed that VEGF signaling affects vascular morphogenesis by controlling the orientation of endothelial cell division perpendicular to the vessel long axis (Zeng et al., 2007). It is reported that this process is affected by a number of factors. First, oriented endothelial divisions appear to be associated with VEGF but is independent of blood flow during early development (Bautch, 2012). Blood vessels derived from embryonic stem cells do not undergo flow-directed endothelial cell division, whose plane of division is perpendicular to the long axis of the vessel, and retinal vessels near the vascular front that likely have low shear stress also orient endothelial divisions (Zeng et al., 2007). Within the vascularized retina, by binding to VEGFR2, VEGF alters the orientation of endothelial cell cleavage planes during anaphase in the major veins and arterioles to further increase vessel tortuosity and dilation independent of eNOS (Hartnett et al., 2008). Therefore, eNOS does not appear to be essential in VEGF-mediated orientation of endothelial cell division.

In the process of forming new blood vessel branches through angiogenesis, endothelial tip cells, which lead nascent vessels (Herbert and Stainier, 2011), likely underpin asymmetric cell division by asymmetric positioning of the mitotic spindle to form asymmetries in cell size and VEGFR signaling components during anaphase (Costa et al., 2017). In this way, the formation of the leading tip and trailing stalk endothelial cells is precisely regulated. Importantly, asymmetries in VEGF signaling following division have been shown to be essential for normal vessel formation by instantly re-establishing the tip-stalk hierarchy and maintaining uninterrupted collective migration during proliferative growth (Costa et al., 2016). Costa et al., confirmed that the larger distal daughter of tip cell division inherited a greater proportion of the VEGF signaling machinery and displayed higher levels of VEGF signaling, establishing it as the leading tip cell. In the absence of differential VEGFR activity the tip-stalk arrangement of daughters was disrupted and cells display symmetric motilities (Costa et al., 2016). We can guess this may explain why abnormal vascular patterns develop in some pathological angiogenesis. Furthermore, in a model of human retinopathy of prematurity (ROP), endothelial NADPH oxidase 4 regulated VEGF receptor (VEGFR)2-mediated angiogenesis and intravitreal neovascularization through activated STAT3 (Wang et al., 2014). However, whether this process further influences angiogenesis by influencing spindle orientation is not clear.

### Natural Direct Current Electric Fields (DC-EFs)

**FIGURE 2:**
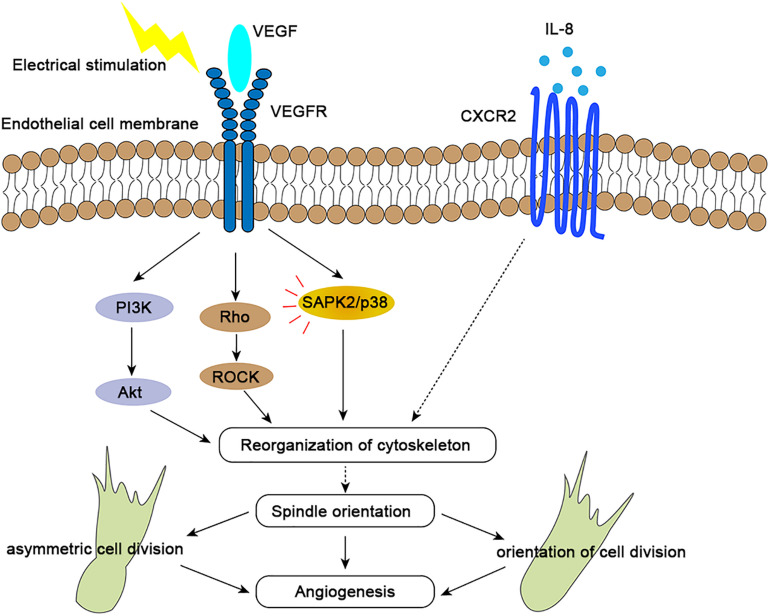
Molecular mechanisms of spindle orientation effects in angiogenesis. By binding to VEGFR2, VEGF activates the PI3K/Akt, Rho/ROCK, and SAPK/p38 signaling pathways (red lines), which results in precise orientation of the mitotic spindle, endothelial cell asymmetry and orientation, and consequent directional angiogenesis. Interleukin-8 (IL-8) binds with high affinity to the chemokine receptor CXCR2, which may also regulate spindle orientation by influencing reorganization of cytoskeleton to further affect spindle orientation, further impacting angiogenesis. Both receptors and signaling pathways are upregulated upon electrical stimulation.

Endogenous electric fields, which have been measured directly in animals and in humans, are ubiquitous and may play a significant role in development (McCaig and Zhao, 1997). Electrical stimulation has emerged as a novel approach to induce angiogenesis *in vivo*, and this process is regulated through increased expression of VEGF in muscle cells (Cuevas and Asin-Cardiel, 2000). DC EFs of 200 mV/mm increased secretion of vascular endothelial growth factor (VEGF) and interleukin 8 (IL-8) in starved HUVEC cells (Zhao et al., 2004; Bai et al., 2011). It has been proved that electric fields of 150–400 mV/mm induced reorientation of the long axis of the endothelial cells perpendicular to the EF vector (Zhao et al., 2004; Cunha et al., 2019). This process were mediated by VEGFR activation, with downstream Rho-ROCK and PI3K-Akt activation leading to cytoskeletal reorganization and the mitotic spindle orientation (Zhao et al., 2004; Figure 2). Electric fields also upregulate the expression of the chemokine receptors CXCR4 and CXCR2 (Cunha et al., 2019). Interleukin 8 (IL-8) with high-affinity binding to the CXCR2 chemokine receptors may stimulate endothelial cell proliferation (Holmes et al., 1991; Baggiolini et al., 1997; Leclair et al., 2014). However, how these chemokines affect orientation of endothelial cell division is still unclear (Figure 2). In general, electrical stimulation may play a spatial organization role in angiogenesis by regulating the endothelial mitotic spindle orientation (Bai et al., 2004).

### G-Protein-Signaling Modulator 2 (GPSM-2 or LGN)

Proper mitotic spindle orientation requires that astral microtubules are connected to the cell cortex by the microtubule-binding protein NuMA. Its cortical recruitment is mediated via direct binding to the adaptor protein LGN which participates in MT-orienting complexes to further regulate mitotic spindle orientation (Yang et al., 2014; Takayanagi et al., 2019). In many cell types, mitotic spindle orientation relies on the canonical “LGN complex” composed of Pins/LGN, Mud/NuMA, and Gαi subunits, which is evolutionary conserved in Drosophila and vertebrates (Saadaoui et al., 2017; Kschonsak and Hoffmann, 2018). In vertebrates, the Gαi-LGN-NuMA complex anchors astral microtubules and orients spindles to regulate asymmetric divisions (Zhu et al., 2011). However, although LGN knockdown perturbs overall endothelial sprouting, spindle orientation in sprouting endothelial cells do not require LGNLGN instead influences interphase microtubule dynamics in endothelial cells to regulate migration, cell adhesion, and sprout extension (Wright et al., 2015). So the potential role of LGN in the blood vessel development may be focused on cell migration and adhesion. In addition, these data might also indicate that different mechanisms regulate spindle orientation in vascular endothelial cells and other cells, and this requires further study. At the same time, there may be novel pathway contributing to spindle orientation during blood vessel formation and therefore possible new therapeutic targets need to be discovered.

### The Rho Family of GTPases: Cdc42

Cdc42, a small GTPase, controls spindle orientation during cell division to regulate epithelial morphogenesis and repair (Jaffe et al., 2008; Mitsushima et al., 2009; Xia et al., 2015). With regards to the development of blood vessels, Cdc42 is essential in embryonic development and a vital regulator of endothelial cell development, regulating actin-based morphogenesis and cell polarity. An absence of Cdc42 results in embryonic death through angiogenesis defects (Jin et al., 2013; Barry et al., 2015). Cdc42 also plays an important role in endothelial regeneration and vascular repair (Flentje et al., 2019). Endothelial cell regeneration is important in the resolution of inflammation and the restoration of vascular integrity after inflammatory vascular injury (Zhao et al., 2014). Cdc42 was shown to affect endothelial cell proliferation through the PAK1/Akt pathway to further regulate vascular recovery after inflammatory lung injury (Lv et al., 2018).

Cdc42 is also involved in sphingosine-1-phosphate (S1P) signal transduction to enhance the barrier function of endothelial monolayers, which can promote vascular stability (Reinhard et al., 2017). It has also been proposed that an absence of Cdc42 leads to defective endothelial axial polarization, sparing endothelial cell proliferation but preventing them from precisely re-distributing within the vascular network and resulting in severe vascular malformations as a consequence of defective cell migration (Laviña et al., 2018). Whether mitotic spindle orientation is of relevance for vascular malformations remains to be further elucidated.

## Spindle Misorientation Is Associated With Diseases

The orientation of the cell division axis determines the positions of daughter cells in a tissue and is therefore crucial to tissue morphogenesis and cell fate decisions (Théry and Bornens, 2006; di Pietro et al., 2016; Chen et al., 2019). Recent studies have shown that a number of factors can regulate the orientation of mitotic spindles and therefore cell division orientation (Li J. et al., 2019). As is stated above, intrinsic factor VEGF signaling and extrinsic factor electric fields (EFs) play an important role in affecting the mitotic spindle orientation to regulate the blood vessel development. An increasing number of vascular development disorders have been reported to result from spindle orientation defects.

Firstly, human retinopathy of prematurity (ROP) has been linked to altered spindle orientation. aberrant mitotic spindle orientation causes ROP by changing endothelial cell cleavage plane and further increasing vessel tortuosity and dilation (Hartnett et al., 2008; Figure 1B). Secondly, diabetic retinopathy (DR), the most common microvascular complication of diabetes maybe also be associated with spindle misorientation. VEGF is overexpressed in hyperglycemic environments and is up-regulated by tissue hypoxia, which increases vessel tortuosity and dilation by altering the orientation of endothelial cell cleavage planes during anaphase in the major veins and arterioles within the vascularized retina (Figure 1B; Hartnett et al., 2008; Capitão and Soares, 2016). Moreover, blood vessels provide nutrients and oxygen to tumors, and insufficient or abnormal angiogenesis contributes to tumor survival, invasion, and metastasis (Saman et al., 2020). In addition to providing nutrients and oxygen to the tumor and the removal of metabolic waste, new vessel formation also enables cancer cells to metastasize and proliferate to distant sites through entry into the newly formed blood and lymphatic system and subsequent extravasation (Nishida et al., 2006). Thus, spindle orientation in the tumor vasculature has become a new key anti-tumor therapeutic target. It is imperative that future studies determine in which of these diseases spindle misorientation contributes to pathogenesis. Beyond that, the mechanisms that prevent spindle misorientation need to be uncovered.

## Conclusion and Perspectives

Cell division orientation plays an essential role in tissue morphogenesis and cell fate decisions. This is achieved through the formation of the mitotic spindle (Lu and Johnston, 2013). The VEGF/VEGFR signaling pathways is key regulator of spindle orientation during angiogenesis (Hartnett et al., 2008), which controlling the orientation of endothelial cell division perpendicular to the vessel long axis to further affect vascular morphogenesis (Zeng et al., 2007). At the same time, IL-8/CXCR2 signaling pathway also was activated during electric fields exposure. Therefore, there has been a hypothetical mechanism that VEGF induces CXCR2 production by endothelial cells, creating a positive-feedback loop to influence spindle orientation (Cunha et al., 2019). Although many of the mechanisms by which planar spindle orientation are tightly regulated and the roles of mitotic spindle orientation in epithelial development and disease have been well studied (di Pietro et al., 2016), further research is needed to see the details of the underlying mechanisms of how spindle orientation affects vascular development by regulating endothelial cell orientation. The differences between the epithelium and endothelium are important to take into consideration. For example, some spindle orientation-related proteins playing important roles in epithelia, such as LGN, did not affect the oriented division of endothelial cell (Wright et al., 2015). Consequently, further studies of the basic molecular mechanisms of how spindle orientation in endothelial cells influences angiogenesis are required, not least to identify potential therapeutic targets. A key challenge will be to determine the precise *in vivo* mechanism of plane spindle orientation and its involvement in blood vessel development.

Aberrant spindle orientation is hypothesized to contribute to tissue disorganization (Qin et al., 2017; Xie et al., 2019). Targeted anti-VEGF therapies have been widely researched, but they cause various side-effects such as hypertension, and are susceptible to drug resistance (Bergers and Hanahan, 2008). Advances in electrical stimulation and an improved understanding of the biological effects of stimulation might lead to new therapies to enhance blood vessel repair and regeneration and to treat diseases or conditions in which angiogenesis is part of the pathogenesis (Cunha et al., 2019). ES has been widely used to induce neurogenic and cardiomyogenic regeneration (Ragnarsson, 2008; Zhang et al., 2011) through regulating endothelial cell migration to wound site (Jeong et al., 2017) and affecting endothelial cell division orientation via VEGF signaling (Zhao et al., 2004). However, spindle orientation proteins have yet to be targeted directly in endothelial cell therapy, and further work is required to establish how mitotic spindles control the orientation and asymmetry of endothelial cells during angiogenesis to leverage the process for the treatment of vascular development-related diseases. Overall, investigation of the role of spindle misorientation in diseases is just beginning, and the most intriguing questions remain to be addressed.

## Author Contributions

XW wrote the manuscript and drew the figures. JZ revised the manuscript. DL conceived the study and edited the manuscript. All authors contributed to the article and approved the submitted version.

## Conflict of Interest

The authors declare that the research was conducted in the absence of any commercial or financial relationships that could be construed as a potential conflict of interest.
